# Contagiousness of Puerperal Fever

**Published:** 1846-03

**Authors:** 


### SELECTIONS

FROM

### AMERICAN AND FOREIGN JOURNALS.

---

*Contagiousness of Puerperal Fever.*—Dr. Kneeland, in the January number of the American Journal of the Medical Sciences, comes to the following conclusions on this point:

1. From the confinement of cases to the practice of single physicians and nurses in populous cities; from the fatal results attending post-mortem examinations; from its ravages in hospitals; that puerperal fever is contagious; that it may have other modes of propagation, in certain states of the atmosphere, and among strongly predisposed individuals; but that the fact of its conveyance by practitioners attests its contagiousness.

2. That it may be propagated by direct inoculation with the fluids of the living and the dead; by the effluvia arisng from the bodies of the sick, inhaled in the very chamber of death, (as in the wards of a hospital,) or carried about by the person of the physician; by clothes, bedding, (fomites,) which have been in contact with a diseased individual.

3. That the order of propagation from the physician to the patient, and the regular succession of cases, show that the epidemics of puerperal fever are, in almost all cases, the *effects* and not the *causes* of the contagion.

4. The contagion acts according to the frequency of communication between the physician or nurse, (in whose practice are cases,) and lying-in women, independently of insalubrity of places, wretchedness of patients, or the neighborhood of dwellings—for, although poverty and misery seem to predispose to it, communication is none the less fatal to the higher classes.

A case, to all appearance sporadic, may communicate the disease; a mild case may, communicate a severe disease, and *vice versa.*

6. Immunity proves nothing against contagion; it may be the effect of an acquired or temporary inaptitude—it is equally inexplicable in all contagious diseases.

7. The rapidity of its propagation shows that it is contagious at the commencement; the fatal results of attending autopsies, indicate this character after death.

8. That a physician should not make, or be present at an autopsy of this disease; or, if he does, should take proper measures to cleanse himself and dress, for the safety of his next patient—that if a case (or several cases) occur in his practice, he should consider himself, in the language of Dr. Holmes, "a private pestilence," and regulate his conduct accordingly—that persons who have washed, or have otherwise handled the clothes or bedding soiled by the discharges of this disease, should not approach, much less nurse, a woman after delivery.

9. That when the disease is prevalent, a prompt removal from possible intercourse with a "pestilential" physician, and a strict attention to ventilation, cleanliness, quiet, proper food, &c., are the dictates of a reasonable fear.—*Amer. Jour. of the Medical Sciences.*

